# Real-world evidence of treatment patterns and survival of metastatic gastric cancer patients in Germany

**DOI:** 10.1186/s12885-024-12204-x

**Published:** 2024-04-13

**Authors:** Jaime Luna, Nils Picker, Thomas Wilke, Magnus Lutz, Jürgen Hess, Bernhard Mörtl, Yan Xiong, Thorsten Oliver Götze

**Affiliations:** 1 Cytel - Real World and Advanced Analytics, Berlin/Wismar, Germany; 2 IPAM, University of Wismar, Wismar, Germany; 3 Daiichi Sankyo Deutschland GmbH, Munich, Germany; 4 Daiichi Sankyo Inc, Basking Ridge, NJ USA; 5 Krankenhaus Nordwest, Frankfurt/Main, Germany; 6 Institut Für Klinische Krebsforschung IKF GmbH Am Krankenhaus Nordwest, Frankfurt/Main, Germany

**Keywords:** Metastatic Gastric Cancer, Real-world evidence, Cohort, Treatment, Survival, Germany

## Abstract

**Background:**

Patients with metastatic gastric cancer (mGC) have poor prognosis. This real-world study aimed to describe treatment regimens and survival of mGC patients.

**Methods:**

A retrospective analysis was conducted using anonymized German claims data (AOK PLUS) covering a period from 2010 to 2021. The study population included newly diagnosed mGC cases identified from 2011 to 2020. The index date was defined as the first diagnosis of metastasis on or after gastric cancer diagnosis. Therapy regimens were identified based on inpatient and outpatient data, and subsequently stratified by line of treatment. Survival analyses were conducted using the Kaplan–Meier method.

**Results:**

The cohort consisted of 5,278 mGC incident cases (mean age: 72.7 years; male: 61.9%). Nearly half of the incident cases received mGC-related treatment (49.8%). Treated patients were more often male, younger, and had fewer comorbidities compared to untreated patients. Of the 2,629 mGC patients who started the first line of treatment (1LOT), 32.8% switched to 2LOT, and 10.2% reached 3LOT. Longer survival time was observed among disease-specific treated cases compared with untreated cases (median real-world overall survival (rwOS): 12.7 months [95%CI 12.1 – 13.3 months] vs. 3.7 months [95%CI 3.4 – 4.0 months]).

**Conclusion:**

Systemic therapy was not received in almost half of the mGC patients. In those patients, a very short median rwOS was observed. Treatment patterns were generally in line with the guideline recommendations, however, therapy switching rates and poor prognosis indicate high unmet needs also in the treated population.

**Supplementary Information:**

The online version contains supplementary material available at 10.1186/s12885-024-12204-x.

## Background

Gastric cancer (GC) is the fourth leading cause of cancer-related mortality worldwide [1] and remains a leading contributor to the global disability-adjusted life-year burden [2]. Incidence progressively increases with age (from around 50 years old) [3]. Considerable differences in risk are observed by sex [4], with males showing higher incidence compared to females [5, 6]. In Germany, the standardized incidence rate was 6.8 per 100,000 females and 14.3 per 100,000 males in 2018, while the standardized mortality rate was 4.1 per 100,000 females and 7.7 per 100,000 males in the same year [7]. For decades, a steady decline in GC incidence and mortality rates has been observed in developed countries, including Germany [7].

In 40% of the cases the disease is already metastasized at the time of Gastric cancer diagnosis [7, 8]. Moreover, disease recurrence is observed in more than 30% of curatively treated patients [9]. Metastatic spread is generally fatal, causing organ compromise and failure of physiological homeostasis [8]. Treatment is mainly selected based on the stage of disease, the presence of biomarkers, and the physician's preferred regimen. In the metastatic setting, first-line treatment may consist of doublet or triplet chemotherapies including taxanes, platinum compounds, and a cytotoxic compound such as 5-fluorouracil with or without trastuzumab (if human epidermal growth factor receptor 2 (HER2) is overexpressed) [10]. Taxanes (docetaxel, paclitaxel), irinotecan or ramucirumab (alone or in combination with paclitaxel) are recommended as second-line treatment options. The landscape in the latter lines of treatment is more challenging in terms of therapeutic algorithms and a poorer prognosis for patients.

The development of targeted therapies is crucial to improve patient care and to address the high unmet need by this innovative approach of treatment. For example, the monoclonal antibody Trastuzumab (target: HER2) has shown an improved response in HER2-positive disease compared to standard systemic chemotherapy [11]. Therefore, HER2 testing is recommended by guidelines in all metastatic GC (mGC) patients [12, 13]. Real-world data offers the opportunity to gain needed insights into the treatment approaches in clinical practice and related outcomes in mGC care. A retrospective analysis of German claims data was conducted aiming to generate evidence on treatment regimens and survival in mGC patients. In addition, HER2-positive mGC patients actively treated with targeted therapy were identified, and relevant outcomes were described.

## Methods

### Study design and data source

A retrospective analysis was conducted using anonymized German claims data provided by AOK PLUS covering the period from January 1, 2010, to December 31, 2021. AOK PLUS, the sixths-biggest German sickness fund, covers approximately 3.5 million insured persons in the regions of Saxony and Thuringia in central-eastern Germany. This corresponds to 50% of the local population and approximately 4.8% of the overall German Statutory Health Insurance (SHI) population. The analyzed dataset comprised anonymized patient-level data on claims for inpatient care, outpatient care, outpatient drug prescriptions, and additional relevant data, including demographic information and all-cause of death.

### Study population

The study population consisted of newly diagnosed mGC cases identified using German Modification of the International Classification of Diseases Version 10 (ICD-10-GM) codes. A metastatic-free period of at least 12 months was established before the index date (to identify mGC incident cases). The index date was defined as the first documented diagnosis of metastasis on or after the date of GC diagnosis. The following selection criteria were applied to retrieve the mGC population:

First, the disease criterion: At least one inpatient diagnosis of GC (ICD-10-GM code: C16.-) and/or at least two confirmed outpatient GC diagnoses in two quarters within 12 months. Second, the metastatic criterion: Any inpatient or confirmed outpatient diagnosis of metastasis (ICD-10-GM codes: C77-80) on or after the date of GC diagnosis. Third, the incident metastatic criterion: A metastatic-free period of at least 12 months before the first observable claim related to metastatic diagnosis. Fourth, the insurance criterion: Continuously insured for at least 12 months prior to the first observable metastatic diagnosis. Fifth, the age criterion: Age ≥ 18 years on the date of the first metastatic diagnosis. Sixth, the non-breast cancer (BC) criterion: No breast cancer diagnosis (ICD-10-GM code: C50.-) before or on the date of the first metastatic diagnosis. Seventh, the non-GIST criterion: No treatment for malignant gastrointestinal stromal tumor with imatinib (Anatomical Therapeutic Chemical Classification System (ATC) code: L01EA01, L01XE01; operation and procedure classification system (OPS) code: 6–001.g) and/or sunitinib (ATC code: L01EX01, L01XE04; OPS code: 6–003.a) during the entire study period. In addition, HER2-positive mGC patients were identified by a treatment proxy [treatment with trastuzumab (ATC code: L01XC03; OPS codes:6–001.7, 6–001.k) at any time after the first observed metastatic diagnosis] for subgroup analyses.

Incident mGC patients were identified during a 10-year period (from 2011 to 2020) by applying the selection criteria. Ensuring a reasonable time to record therapy treatments and time-to-event outcomes, data were considered until December 31, 2021. An overview of the study period and inclusion period is shown in Supplementary Fig. 1.

### Treatment line definitions

mGC therapies were identified through inpatient and outpatient codes. The treatment codes are listed in Supplementary Table 1. The first line of treatment (1LOT) was established considering the first prescription/application of any of the agents listed in Supplementary Table 1. All therapies documented within 28 days after starting a LOT were considered part of combination therapy. A new line of treatment was initiated if an additional active substance or combination of substances (that was not part of the current LOT) was prescribed after 28 days or more or if therapy was restarted after a gap of more than three months. When an unspecified OPS code was documented for inpatients (chemotherapy with unspecified substances code, OPS 8–54), it was assumed that the preceding LOT was continued during the period of hospitalization. No new treatment line was established for switching between platinum compounds (carboplatin, cisplatin, or oxaliplatin).

### Statistical analysis

Qualitative variables were described as frequencies with percentages, and quantitative variables as mean ± standard deviation (SD). Baseline characteristics of mGC patients were stated at time of first mGC diagnosis, including age, sex, comorbidities identified through ICD-10 codes (Charlson Comorbidity Index (CCI) score [14], Supplementary Table 2), and the number of all-cause hospitalizations.

The most frequent therapy regimens were described numerically overall and by treatment line. Real-world time-to-treatment initiation (rwTTI) and real-world time-to-next-treatment (rwTTNT) were assessed using Kaplan–Meier (KM) method. RwTTI was estimated from mGC diagnosis until the date of death or censoring at end of continuous insurance or end of the study, whichever occurs first. RwTTNT was estimated since the start of each treatment line among those who reached the next LOT.

Survival analyses were conducted from the date of mGC diagnosis until the date of death (all-cause) or censoring at end of continuous insurance or end of the study, whichever occurs first. KM method was used to estimate real-world overall survival (rwOS). Median survival time and survival probabilities were reported. KM curves were displayed along with the number of patients at risk. Analyses were performed in each treatment line and in the HER2-positive mGC patients subgroup.

All findings were reported using the guidelines of the Strengthening the Reporting of Observational Studies in Epidemiology (STROBE) [15] and Standardized Reporting of Secondary data Analyses (STROSA) [16]. Descriptive and analytical statistical analyses were conducted using STATA software version 17 (STATA Corp., College Station, Texas).

## Results

**Fig. 1:**
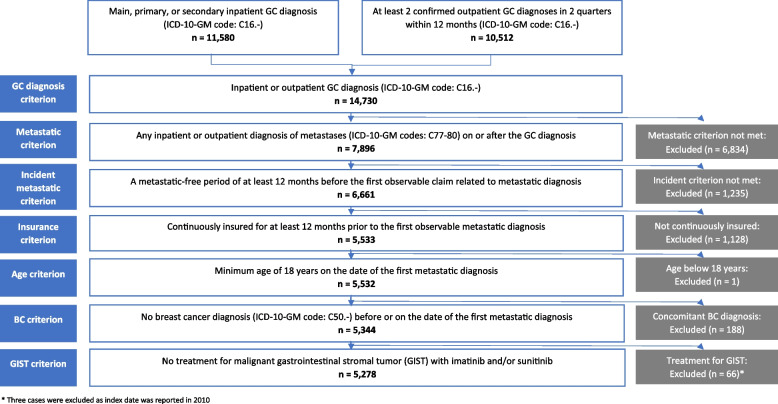
Attrition chart of patient selection

Overall, 14,730 GC cases were identified in the inclusion period. After eligibility criteria were applied, 5,278 mGC were included. The attrition chart of the selection process is shown in Fig. 1.

### Patient characteristics

The cohort consisted of 5,278 mGC newly diagnosed cases. The mean age at the time of diagnosis was 72.7 years (SD 11.6). A predominance of male cases was observed (61.9%). Table 1 shows the baseline characteristics of mGC patients. 214 HER2-positive mGC patients were identified using a treatment proxy (trastuzumab) which represents 4.1% of the mGC cohort. Baseline characteristics of HER2-positive mGC patients are described in Supplementary Table 3.

**Table 1:** Patient characteristics of the mGC cohort overall and stratified by disease-specific untreated, disease-specific treated, and lines of treatment

	**mGC**^**c**^ **(*n* = 5,278)**	**Disease-specific untreated**^**c**^ **(*n* = 2,649)**	**Disease-specific treated**^**c**^ **(*n* = 2,629)**	**1LOT**^**d**^ **(*n* = 2,629)**	**2LOT**^**d**^ **(*n* = 861)**	**3LOT**^**d**^ **(*n* = 268)**
**Age**, *mean (SD)*	72.7 (11.6)	77.4 (10.2)	68.1 (11.0)	68.3 (11.0)	66.8 (11.3)	66.3 (11.5)
**Sex**						
*Female, n (%)*	2,013 (38.1)	1,126 (42.5)	887 (33.7)	887 (33.7)	259 (30.1)	78 (29.1)
*Male, n (%)*	3,265 (61.9)	1,523 (57.5)	1,742 (66.3)	1,742 (66.3)	602 (69.9)	190 (70.9)
**Charlson Comorbidity Index score**^a^*, mean (SD)*^b^	3.0 (2.5)	3.7 (2.6)	2.3 (2.2)	2.4 (2.2)	2.2 (2.1)	1.9 (2.0)
**Number of hospitalizations**^**a**^***,**** mean (SD)*	2.0 (1.9)	2.1 (1.8)	1.9 (2.0)	2.6 (2.0)	4.6 (4.6)	3.3 (3.0)

^a^12-month period pre-index date or pre-starting the treatment line (incl. index date or start date for LOT); ^b^Metastatic solid tumor diagnosis at index (ICD-10: C77-C80) and gastric cancer (ICD-10: C16) diagnosis were excluded for the CCI score; ^c^Patients characteristics at time of diagnosis; ^d^Patients characteristics at the start of each line of treatment

Around half of the newly diagnosed mGC cases received a disease-specific treatment regime (49.8%). Compared to disease-specific untreated cases, treated cases were younger (treated: 68.1 years vs. untreated: 77.4 years), represented a higher proportion of male cases (treated: 66.3% vs. untreated: 57.5%), had lower CCI (mean: treated 2.3 vs. untreated 3.7) and lower number of hospitalizations within the 12-month pre-index period (mean: treated 1.9 vs. untreated 2.1; Table 1).

### Treatment patterns

2,629 mGC cases started 1LOT. Of those patients, 32.8% switched to 2LOT, and 10.2% reached 3LOT. The median rwTTI among treated was 0.9 months (95%CI 0.8 – 0.9 months). The median rwTTNT from 1 to 2LOT was 7.3 months (95%CI 6.9 – 7.7. months) and 6.3 months (95%CI 5.3 – 7.4 months) from 2 to 3LOT. rwTTNT in HER2-positive mGC patients is described in Supplementary Table 4.

**Fig. 2:**
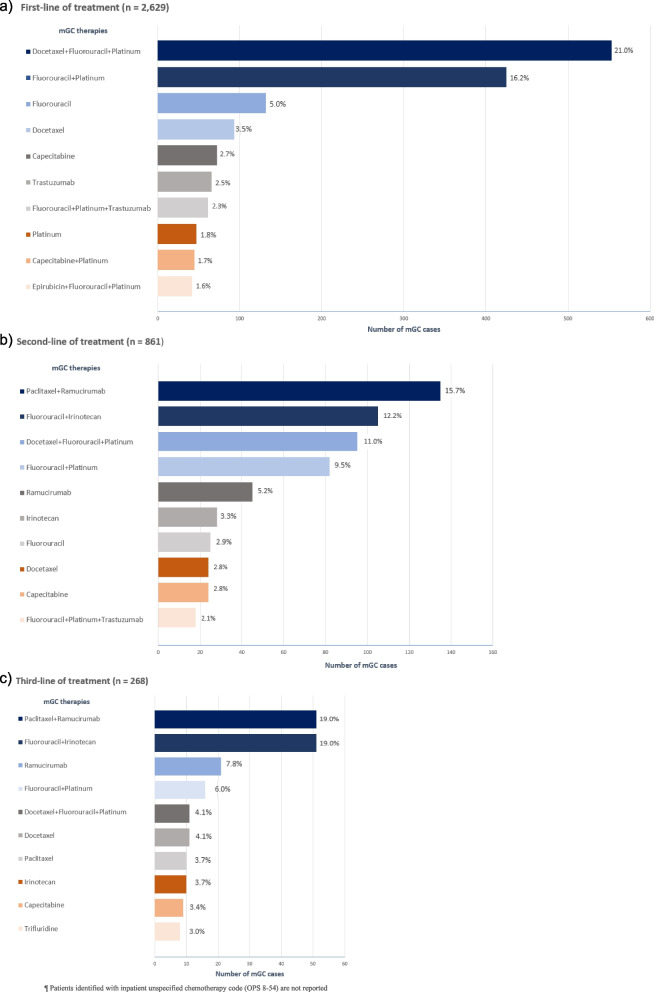
The most frequently mGC therapy regimens from 2011 stratified by line of treatment: (**a**) first-line of treatment, (**b**) second-line of treatment, (**c**) third-line of treatment

The most frequent treatment regimen used in 1LOT was docetaxel + fluorouracil + platinum compound (21.0%), followed by fluorouracil + platinum compound (16.2%). Paclitaxel + ramucirumab (15.7%), fluorouracil + irinotecan (12.2%), and docetaxel + fluorouracil + platinum compound (11.0%) were the most frequently prescribed combinations in 2LOT, while fluorouracil + irinotecan (19.0%) and paclitaxel + ramucirumab (19.0%) were mostly identified in 3LOT. Nevertheless, inpatient chemotherapy with unspecified substances was found in 29.9% for 1LOT, 12.0% for 2LOT, and 4.1% for 3LOT. The most frequent mGC therapy regimens used from 2011 are shown in Fig. 2. To provide a current treatment perspective, the mGC therapy regimens from 2016 are described in Supplementary Table 5.

### Real-world overall survival

**Fig. 3:**
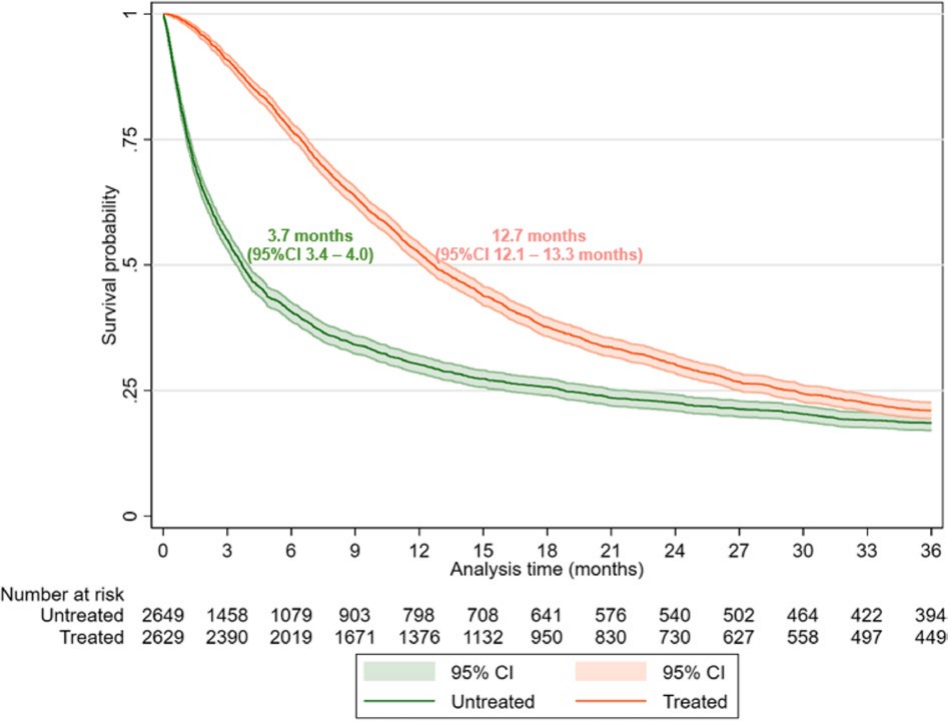
Kaplan–Meier survival curves for patients with metastatic gastric cancer since diagnosis. Kaplan–Meier curves: real-world overall survival between disease-specific treated and disease-specific untreated in mGC cases

**Fig. 4:**
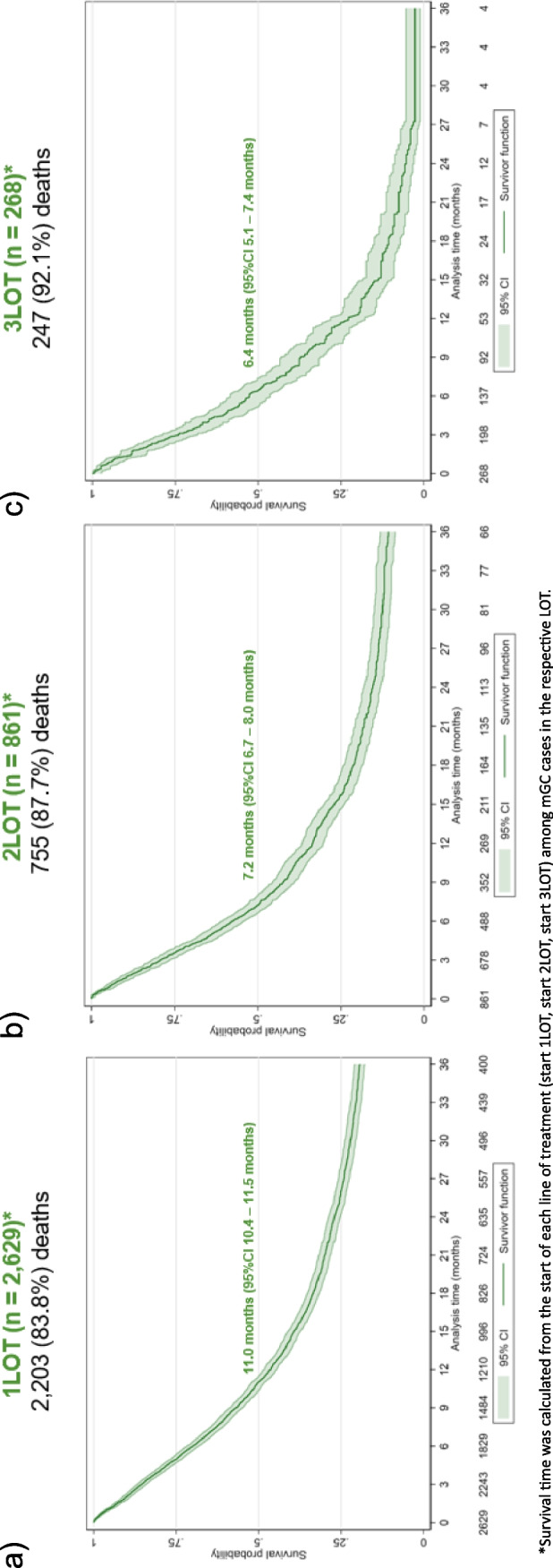
Kaplan–Meier survival curves for patients with metastatic gastric cancer since the start of line of treatment. Kaplan–Meier curves: (a) real-world overall survival since the start of 1LOT, (b) real-world overall survival since the start of 2LOT, (c) real-world overall survival since the start of 3LOT

4,481 (84.9%) patients had already died at the time of data cut-off. The median rwOS since diagnosis of metastasis was 8.5 months (95%CI 8.1 – 9.0 months). The survival probability was 41.1% (95%CI 39.8%—42.5%) at 1 year and 14.1% (95%CI 13.1%—15.2%) at 5 years. Longer survival time was observed among disease-specific treated cases compared with untreated cases (median rwOS: 12.7 months, [95%CI 12.1 – 13.3 months] vs. 3.7 months [95%CI 3.4 – 4.0 months]; Fig. 3). Figure 4 shows survival time since the start of each treatment line (median rwOS: 11.0 months [95%CI 10.4 – 11.5 months] from the start of 1LOT, 7.2 months [95%CI 6.7 – 8.0 months] from the start of 2LOT, and 6.4 months [95%CI 5.1 – 7.4 months] from the start of 3LOT).

Among HER2-positive mGC cases, 190 (88.8%) deaths were reported. The median rwOS since diagnosis of metastases was 16.1 months (95%CI 13.2 – 17.5 months; Supplementary Fig. 2). The survival rate was 61.2% (95%CI 54.3%—67.4%) at 1 year and 10.2% (95%CI 6.4%—15.0%) at 5 years.

## Discussion

Our real-world study provides key data to assess the disease burden of mGC in Germany, which is highly relevant to support discussing healthcare planning and the allocation of resources. The findings on treatment patterns offer a clear picture of the therapy regimens and the sequential switching of treatment lines in clinical practice. Survival analysis showed that mGC remains a life-threatening disease, for which new treatment options are needed to improve prognosis.

Almost half of mGC patients remained without systemic therapy. Patients who received disease-specific therapy were more likely to be male, young, and had fewer comorbidities. Palliative care can effectively relieve symptoms as well as improve the quality of life [17]. The decision to offer supportive care alone or with systemic therapy is dependent on the patient's performance status [13]. It is necessary to individualize the approach, for instance in patients with a late diagnosis and poor prognosis. Other real-world studies have reported a relevant proportion of mGC without systemic therapy. For instance, a study using the Flatiron Health database showed that one-quarter of patients with mGC did not receive treatment and only approximately one-half of patients receiving 1LOT received subsequent treatment [18]. Furthermore, the proportion of untreated patients in our findings is consistent with the estimations of a retrospective observational study described in the Trifluridin/Tipiracil (Lonsurf®) dossier in Germany [19].

Treatment patterns observed in this study were generally in line with the guideline recommendations and the literature [13, 20, 21]. In the first line of treatment, the two most frequent combinations used were the triplet chemotherapy (docetaxel + fluorouracil + platinum compound) and the doublet chemotherapy (fluorouracil + platinum compound). Both combinations are existing treatment options in this setting according to the German S3 gastric cancer guideline [20]. In second and later-line treatment, ramucirumab-paclitaxel, ramucirumab monotherapy, or irinotecan were frequently prescribed. Here, a longitudinal analysis of treatment patterns in mGC is presented to understand the current therapeutic landscape. This is in contrast to previous studies that are restricted to a specific line of treatment [22–24].

The presented estimates for survival appear to be consistent with the literature [25–28]. The Munich cancer registry (TRM) showed a median OS of 7.3 months for German mGC patients (Stage IV) [9]. The ToGA trial showed that trastuzumab combined with chemotherapy offers a benefit for patients with HER2-positive advanced gastric cancer. The median OS was 13.8 months (95% CI 12 months to 16 months) in those assigned to trastuzumab with chemotherapy compared with 11.1 months (95% CI 10 months to 13 months) in those assigned to chemotherapy alone [11].

For the interpretation of rwOS since the start of therapy lines, it is necessary to consider that at the start of each treatment line, a specific subgroup of patients was analyzed (i.e., only those who were able to reach the further line of treatment).

Our study relies on certain strengths. First, the use of a structured administrative database for insurance claims considering a large sample size. Second, the long study period allowed us to include patients for a decade along with record treatments, interventions, and outcomes of interest during follow-up. Third, data was independently collected in terms of inpatient/outpatient diagnoses and complete records of outpatient prescriptions. It is, however, necessary to recognize that there are general limitations related to the use of claims data. First, our study aimed to describe therapies and survival in mGC patients, while also providing insights on HER2-positive mGC patients actively treated with targeted therapy. Consequently, mGC patients were identified excluding BC patients given that HER-2 is overexpressed in approximately 20% of BC patients and has been associated with poorer prognosis. We also excluded GIST patients with unresectable or metastatic disease using tyrosine kinase inhibitors as a treatment proxy for identification. The exclusion of these patients needs to be considered for the interpretation of the study. Second, clinical and laboratory data are not available in German claims data, which limited our capacity to identify the metastatic stage based on clinical assessment or determine HER2 status. For that reason, a specific algorithm was implemented, and a treatment proxy was used to mitigate these issues. Considering the latest, it is important to mention that HER2-positive patients without targeted treatment were not included in the subgroup analysis. Therefore, HER2-positive analysis described only patients treated with targeted therapy. Third, due to the inpatient chemotherapy with unspecified substances code available in Germany, it was not possible to identify specific treatments for a proportion of patients in the hospital setting. It is also important to consider the assumptions applied in the algorithm to define treatment lines for the interpretation of results. Among other things, it should be noted that combination regimens were only identified as such if the associated drugs were prescribed within a time window of 28 days, since a later prescription would otherwise be identified as a change in the line of therapy. Furthermore, claims databases contain data from routine practice for reimbursement purposes. Despite this, coding is generally considered to be of high quality in these databases. Fourth, patient selection and representativeness of the study are limited to subjects who are insured with German statutory health insurance in the study area. Nevertheless, uniform healthcare regulations with nearly universal coverage and access to health resources across Germany reduce concerns of possible bias, since around 88% of the German population is insured under the SHI system. Lastly, data from routine practice may exhibit coding errors or missing data.

## Conclusion

Systemic therapy was not received in almost half of the mGC cases. In those patients without therapy, a very short median real-world OS (less than 4 months) was observed. Further studies need to determine if patients without systematic treatment have a short survival related to their medical status or the fact that they are not receiving systematic treatment. The documented real-world treatment patterns give a clear picture of the regimens used in clinical practice and confirmed that they were consistent with the national guidelines. However, the high therapy switching rates and the poor prognosis in terms of survival indicate the high unmet needs also in the treated population. The generated information on therapy regimens and survival in GC-care is important for health policymaking to reduce the burden of these patients. Furthermore, our findings represent essential data for establishing new approaches to improve management and survival in mGC patients.

### Supplementary Information

**Supplementary Material 1.**

**Supplementary Material 2.**

**Supplementary Material 3.**

**Supplementary Material 4.**

**Supplementary Material 5.**

**Supplementary Material 6.**

**Supplementary Material 7.**

## Data Availability

The data that support the findings of this study are available from AOK PLUS but restrictions apply to the availability of these data, which were used under license for the current study, and so are not publicly available.
