# Supplementary material for: Real-world evidence of treatment patterns and survival of metastatic gastric cancer patients in Germany
Source: BMC Cancer. 2024 Apr 13;24:462. doi: 10.1186/s12885-024-12204-x (PMC11016202; doi:10.1186/s12885-024-12204-x)
Supplement: Supplementary file 1 — Supplementary Material 1. [file 12885_2024_12204_MOESM1_ESM.pdf]

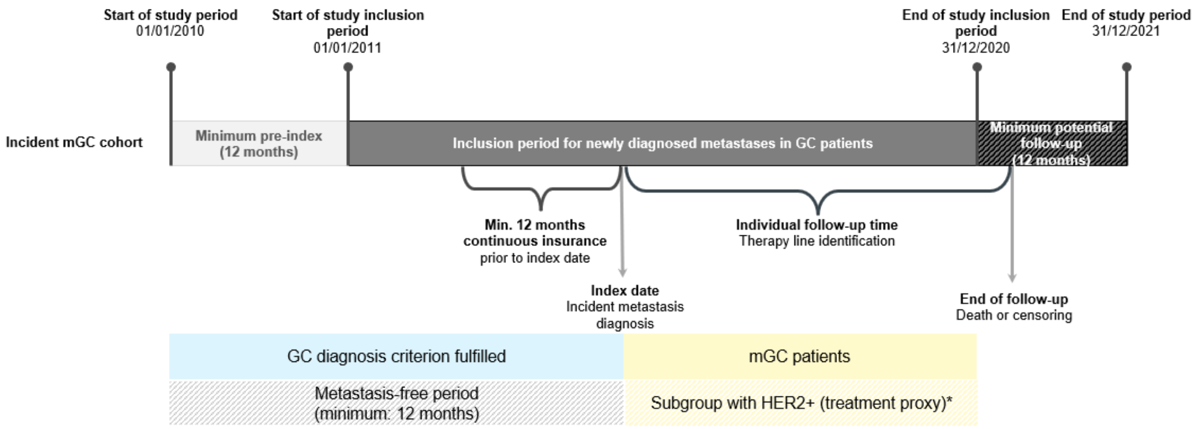

*It needs to be noted that due to the treatment proxy applied to identify HER2+ patients this study does not cover untreated cases (HER2+ without trastuzumab treatment) and therefore the numbers are generally underestimating the HER2+ mGC incidence/prevalence.
