# Supplementary figures and images for: Real-world evidence of treatment patterns and survival of metastatic gastric cancer patients in Germany
Source: BMC Cancer. 2024 Apr 13;24:462. doi: 10.1186/s12885-024-12204-x (PMC11016202; doi:10.1186/s12885-024-12204-x)

a)

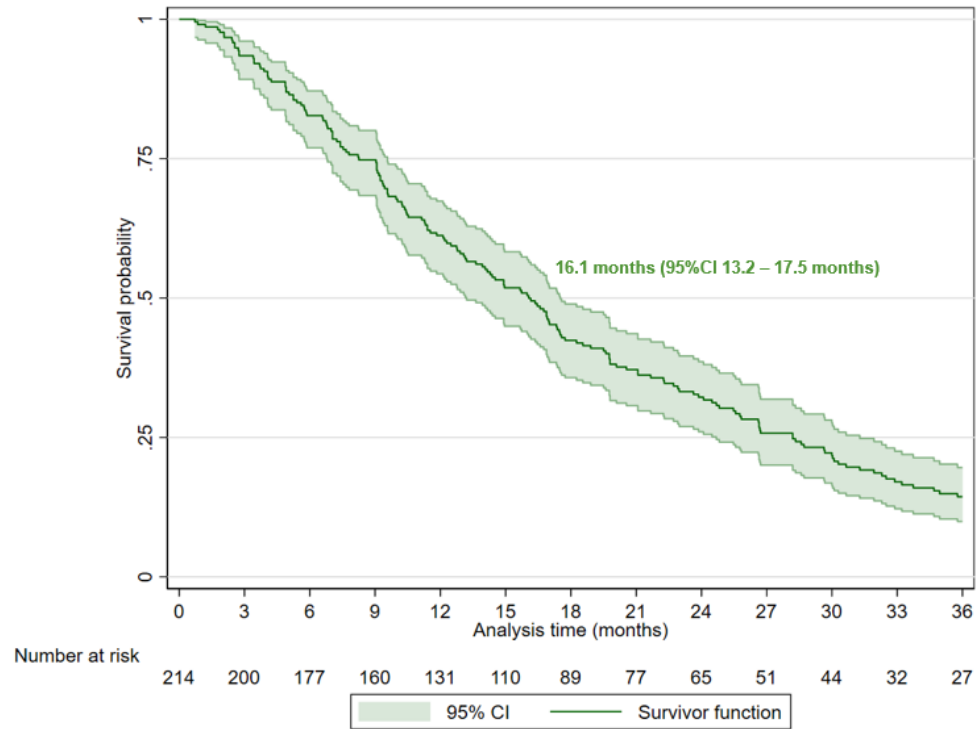

Supplement: Supplementary file 2 — Supplementary Material 2. [file 12885_2024_12204_MOESM2_ESM.pdf]
