# Supplementary material for: Real-world evidence of treatment patterns and survival of metastatic gastric cancer patients in Germany
Source: BMC Cancer. 2024 Apr 13;24:462. doi: 10.1186/s12885-024-12204-x (PMC11016202; doi:10.1186/s12885-024-12204-x)
Supplement: Supplementary file 3 — Supplementary Material 3. [file 12885_2024_12204_MOESM3_ESM.docx]

| **Supplementary Table 1. List of mGC therapies in German inpatient and outpatient codes** | | |
| --- | --- | --- |
| **Treatments used in mGC** | **ATC** | **OPS** |
| Fluorouracil | L01BC02 | N/A |
| Oxaliplatin^[[1]](#footnote-1)^ | L01XA03 | N/A |
| Tegafur (Teysuno) containing therapies | L01BC53 [since 01/01/2010]  L01BC03 [since 01/01/2010]  L01BC73 [since 01/01/2013]  L01BC63 [since 01/01/2014] | N/A |
| Docetaxel | L01CD02 | 6-002.h |
| Paclitaxel | L01CD01 | 6-001.f |
| Ramucirumab | L01XC20 [01/01/2016-31/12/2016]  L01XC21 [01/01/2017-31/12/2021]  L01FG02 [since 01/01/2022] | 6-007.m [since 01/01/2016] |
| Trastuzumab | L01XC03 [until 31/12/2021]  L01FD01 [since 01/01/2022] | 6-001.7 [until 31/12/2014]  6-001.k [since 01/01/2015] |
| Cisplatin | L01XA01 | N/A |
| Capecitabine | L01BC06 | N/A |
| Epirubicin | L01DB03 | N/A |
| Irinotecan | L01XX19 [until 31/12/2020]  L01CE02 [since 01/01/2021] | 6-001.3 |
| Carboplatin | L01XA02 | N/A |
| Pembrolizumab | L01XC18 [until 31/12/2021]  L01FF02 [since 01/01/2022] | 6-009.3 [since 01/01/2017] |
| Mitomycin | L01DC03 | N/A |
| Trifluridine/Tipiracil | L01BC59 [since 01/01/2016] | 6-009.n [since 01/01/2018] |
| Nivolumab | L01XC17 [until 31/12/2021]  L01FF01 [since 01/01/2022] | 6-008.m [since 01/01/2017] |
| Inpatient chemotherapy with unspecified substance | Instillation of and locoregional therapy with cytotoxic materials and immunomodulators   - Into pleural cavity - Intraperitoneal | 8-541-2, 8-541-3 |
|  | Non-complex chemotherapy | 8-542 |
|  | Moderately complex and intensive block chemotherapy | 8-543 |
|  | Highly complex and intensive block chemotherapy | 8-544 |
|  | Hyperthermic chemotherapy | 8-546 |

1. OPS code (6-001.5) excluded from the catalog since 2011 [↑](#footnote-ref-1)
