# Supplementary material for: Real-world evidence of treatment patterns and survival of metastatic gastric cancer patients in Germany
Source: BMC Cancer. 2024 Apr 13;24:462. doi: 10.1186/s12885-024-12204-x (PMC11016202; doi:10.1186/s12885-024-12204-x)
Supplement: Supplementary file 4 — Supplementary Material 4. [file 12885_2024_12204_MOESM4_ESM.docx]

| **Supplementary Table 2. Computation of the Charlson Comorbidity Index (CCI)** | | | |
| --- | --- | --- | --- |
| **No** | **Comorbidity** | **Charlson Score** | **ICD-10 Codes** |
| 1 | Coronary artery disease | 1 | I20.-, I21.-, I22.-, I23.-, I24.-, I25.- |
| 2 | Congestive heart failure | 1 | I11.-, I50.- |
| 3 | Peripheral vascular disease | 1 | I73.-, I74.-, I77.- |
| 4 | Cerebrovascular disease | 1 | G45.-, G46.-, I6.- |
| 5 | Dementia | 1 | F00.-, F01.-, F02.-, F03.-, G30.- |
| 6 | Chronic pulmonary disease | 1 | J4.-, J6.- w/o J67.-, J68.-, J69.- |
| 7 | Connective tissue disorder | 1 | M05.-, M06.-, M07.-, M08.-, M3.- |
| 8 | Peptic ulcer disease | 1 | K25.-, K26.-, K27.-, K28.- |
| 9 | Mild liver disease | 1 | B18.-, K70.-, K73.-, K75.- |
| 10 | Diabetes mellitus without complications | 1 | E109.-, E119.-, E129.-, E139.-, E149.- |
| 11 | Hemiplegia | 2 | G81.-, G82.- |
| 12 | Moderate or severe renal disease | 2 | N17.-, N18.-, N19.- |
| 13 | Diabetes mellitus with end-organ damage | 2 | E10.-, E11.-, E12.-, E13.-, E14.- w/o [No 10] |
| 14 | Tumor without metastases, leukemia, lymphoma, multiple myeloma | 2 | C% w/o [No 16] |
| 15 | Moderate or severe liver disease | 3 | K72.-, K74.-, I85.- |
| 16 | Metastatic solid tumor* | 6 | C77.-, C78.-, C79.-, C80.- |
| 17 | Human immunodeficiency virus [HIV] disease | 6 | B20.-, B21.-, B22.-, B23.-, B24.- |
| 18 | Age factor (was excluded from index) | For each decade ≥50 years of age, 1 point was added to the score | |
| * Metastatic solid tumor diagnosis at index (ICD-10: C77-C80) and gastric cancer (ICD-10: C16) diagnosis were excluded for the CCI score | | | |
