# Supplementary material for: Real-world evidence of treatment patterns and survival of metastatic gastric cancer patients in Germany
Source: BMC Cancer. 2024 Apr 13;24:462. doi: 10.1186/s12885-024-12204-x (PMC11016202; doi:10.1186/s12885-024-12204-x)
Supplement: Supplementary file 5 — Supplementary Material 5. [file 12885_2024_12204_MOESM5_ESM.docx]

| **Supplementary Table 3. Baseline characteristics of HER2-positive mGC patients** | |
| --- | --- |
| **Patient characteristics** | **HER2-positive mGC**  **(n = 214)** |
| **Age**, *mean (SD)* | 67.4 (11.7) |
| **Sex** |  |
| *Female, n (%)* | 55 (25.7) |
| *Male, n (%)* | 159 (74.3) |
| **Charlson Comorbidity Index score****, mean (SD)*** | 2.2 (2.2) |
| **Number of hospitalizations****, mean (SD)* | 1.6 (1.5) |
| *12-month pre-index period (incl. index date); ** Metastatic solid tumor diagnosis at index (ICD-10: C77-C80) and gastric cancer (ICD-10: C16) diagnosis were excluded for the CCI score | |
