# Supplementary material for: Real-world evidence of treatment patterns and survival of metastatic gastric cancer patients in Germany
Source: BMC Cancer. 2024 Apr 13;24:462. doi: 10.1186/s12885-024-12204-x (PMC11016202; doi:10.1186/s12885-024-12204-x)
Supplement: Supplementary file 6 — Supplementary Material 6. [file 12885_2024_12204_MOESM6_ESM.docx]

| **Supplementary Table 4. Real-world time-to-next-treatment in HER2-positive mGC patients** | | |
| --- | --- | --- |
|  | **From 1LOT to 2LOT** | **From 2LOT to 3LOT** |
| **Median rwTTNT in months, (95% CI)** | 7.0 months (5.5 to 8.9 months) | 4.8 months (3.9 – 7.7 months) |
