# Supplementary material for: Real-world evidence of treatment patterns and survival of metastatic gastric cancer patients in Germany
Source: BMC Cancer. 2024 Apr 13;24:462. doi: 10.1186/s12885-024-12204-x (PMC11016202; doi:10.1186/s12885-024-12204-x)
Supplement: Supplementary file 7 — Supplementary Material 7. [file 12885_2024_12204_MOESM7_ESM.docx]

| **Supplementary Table 5. Most frequent therapy regimens by lines of treatment in mGC patients starting a treatment from 2016** | | | | | |
| --- | --- | --- | --- | --- | --- |
| **Therapy regimes in 1LOT** | | **Therapy regimes in 2LOT** | | **Therapy regimes in 3LOT** | |
| **1LOT ^¶^**  **(n = 1,258)** | **n (%)** | **2LOT** ^¶^  **(n = 403)** | **n (%)** | **3LOT** ^¶^**^*^**  **(n = 132)** | **n (%)** |
| Docetaxel + Fluorouracil + Platinum | 354 (28.1) | Paclitaxel + Ramucirumab | 104 (25.8) | Paclitaxel + Ramucirumab | 35 (26.5) |
| Fluorouracil + Platinum | 198 (15.7) | Fluorouracil + Platinum | 44 (10.9) | Fluorouracil + Irinotecan | 22 (16.7) |
| Fluorouracil | 50 (4.0) | Docetaxel + Fluorouracil + Platinum | 42 (10.4) | Ramucirumab | 14 (10.6) |
| Trastuzumab | 41 (3.3) | Fluorouracil + Irinotecan | 40 (9.9) | Trifluridine/Tipiracil | 8 (6.1) |
| Ramucirumab | 30 (2.4) | Ramucirumab | 35 (8.7) | Fluorouracil + Platinum | 7 (5.3) |
| Fluorouracil + Platinum + Trastuzumab | 27 (2.2) | Capecitabine | 11 (2.7) | Irinotecan | 6 (4.6) |
| Capecitabine | 25 (2.0) | Fluorouracil | 10 (2.5) |  |  |
| Paclitaxel + Ramucirumab | 24 (1.9) | Irinotecan | 9 (2.2) |  |  |
| Capecitabine + Platinum | 20 (1.6) | Nivolumab | 9 (2.2) |  |  |
| Platinum | 20 (1.6) | Trastuzumab | 9 (2.2) |  |  |
| ¶ Patients identified with inpatient unspecified chemotherapy code (OPS 8-54) are not reported  *Categories with less than 5 cases were not reported | | | | | |
